# Recurrent severe hypophosphatemia following intravenous iron administration

**DOI:** 10.1002/ccr3.2595

**Published:** 2020-01-09

**Authors:** Melissa Stephanie Nataatmadja, Ross Francis

**Affiliations:** ^1^ Renal Services Sunshine Coast University Hospital and Health Service Birtinya Australia; ^2^ Faculty of Medicine University of Queensland Brisbane Australia; ^3^ Department of Nephrology Princess Alexandra Hospital Brisbane Australia

**Keywords:** hypophosphatemia, intravenous iron, iron infusion, osteomalacia, phosphorous

## Abstract

Hypophosphatemia postintravenous iron is frequent but under‐recognized. If prolonged or recurrent, it can cause osteomalacia. The likely mechanisms are direct toxicity to proximal tubular cells causing phosphate wasting, elevated Fibroblast growth factor‐23 (FGF‐23), and reduced 1,25‐dihydroxyvitamin D (1,25(OH)_2_D). Hypophosphatemia may be severe and persist for months, necessitating phosphate replacement until normalization of serum levels occurs.

## Graphical abstract

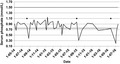

## 1 INTRODUCTION

Intravenous iron is widely used, both in hospital and community settings, and is prescribed by doctors from a wide range of disciplines, including primary care. It is considered a safe and better‐tolerated alternative to oral iron, due to the lack of gastrointestinal side effects.^1^, ^2^ Though the immediate risks of intravenous iron, such as allergic reaction, are generally well‐recognized by prescribing practitioners,^3^ delayed adverse effects may often not be considered.

Even though hypophosphatemia following iron infusion is well reported in the literature, it is under‐recognized in clinical practice. In the majority of reported cases, patients were asymptomatic but, in some cases, patients experienced profound hypophosphatemia and associated symptoms. Repeated iron infusions and associated hypophosphatemia in the long term can result in osteomalacia, so it is important to monitor phosphate levels until they are maintained within the normal range and to supplement phosphate if there is severe, prolonged or symptomatic hypophosphatemia.

## 2 CASE PRESENTATION

A 36‐year‐old kidney transplant recipient was administered 500 mg intravenous iron polymaltose for iron deficiency anemia and intolerance of oral iron supplements. She had a past medical history of end‐stage kidney disease of uncertain etiology and received a kidney transplant two years earlier. Her allograft function was excellent with a serum creatinine of 76 µmol/L. Other past medical history included post‐transplant diabetes managed with insulin and metformin, and osteopenia treated with cholecalciferol and risedronate. A routine blood test performed one week after the intravenous iron infusion showed a very low serum phosphate of 0.20 mmol/L (0.75‐1.50). On review, she described generalized muscle weakness. Initially, the phosphate result was considered a possible factitious result, as her prior serum phosphates had within the normal range; however, repeat testing was congruent. Serum calcium, parathyroid hormone, and vitamin D levels were unremarkable. She was commenced on oral phosphate supplements and both the hypophosphatemia and muscle weakness normalized within a week.

## 3 OUTCOME AND FOLLOW‐UP

**Figure 1:**
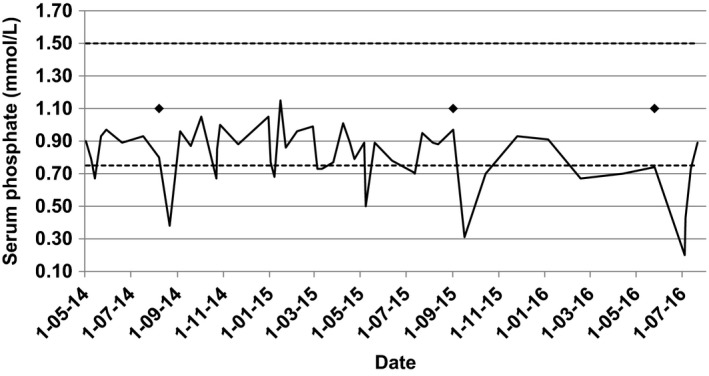
Serum phosphate levels over time with relation intravenous iron infusions (indicated by ♦)

This incident prompted a review of her past medical records and it was observed that she had also experienced hypophosphatemia following previous iron polymaltose infusions (Figure 1). At the time of receiving her first iron infusion approximately two years earlier, her serum phosphate fell from 0.80 to 0.38 mmol/L within two weeks. When rechecked a further two weeks later, the phosphate had recovered to 0.96 mmol/L. Approximately one year later she received another iron infusion. Again, the serum phosphate dropped within 2 weeks, from 0.97 to 0.31 mmol/L, recovering to 0.70 mmol/L four weeks later.

## 4 DISCUSSION

Many drugs are known to cause acute hypophosphatemia, including parenteral nutrition, calcineurin inhibitors, insulin, phosphate‐binding antacids, dopamine, adrenaline, bisphosphonates, theophylline, corticosteroids and diuretics, and this may occur via a number of mechanisms such as extra to intracellular phosphate shifts, decreased intestinal phosphate absorption, increased urinary phosphate extraction as well as pseudohypophosphatemia.^4^

Intravenous iron was first reported to cause hypophosphatemia in 1983 by Okada and colleagues.^5^ Since then, there have been several further reported cases, particularly in Japan where the use of saccharated ferric oxide preparations has been popular since the 1960s and where there have also been several reported cases of hypophosphatemic osteomalacia caused by prolonged intravenous iron use.^6^, ^7^ In order to form a colloidal solution for intravenous use, iron must be complexed with a carbohydrate ligand, which may consist of dextran, sucrose, gluconate or polymaltose, and hypophosphatemia has been reported following the use of most of these types.^8^

Shimizu and colleagues reported a case series of saccharated ferric oxide induced hypophosphatemia and osteomalacia.^6^ One patient was a 43‐year‐old female who had been receiving saccharated ferric oxide for more than 10 years due to chronic iron deficiency anemia from menorrhagia and presented with hypophosphatemia and bone pain. The second case was a female who had also been receiving long term iron infusions, but developed bone pain and hypophosphatemia only after she switched from chondroitin sulfate‐iron to saccharated ferric oxide, whereas the third case was observed to have an acutely declining serum phosphate while an inpatient in hospital receiving saccharated ferric oxide infusions. The authors found that FGF‐23 was elevated in all three patients at the time they were hypophosphatemic and this progressively declined over time. In addition, 1,25‐dihydroxyvitamin D (1,25(OH)_2_D) was inappropriately low and, in all cases, cessation of the saccharated ferric oxide resulted in a return to normophosphatemia, and the use of dextrin citrate‐iron (III) complex did not result in low serum phosphate levels.

In a prospective study by Schouten and colleagues, 8 patients were given intravenous iron polymaltose and followed up with serial biochemistry and urinary phosphate measurements for at least three weeks.^8^ The authors demonstrated a significant decline in serum phosphate as well as reduced tubular phosphate reabsorption from baseline to 1 week, which remained significantly low at weeks 2 and 3. In a few of the cases, the hypophosphatemia lasted as long as 6 weeks postinfusion but did eventually normalize. In this study, FGF‐23 was also significantly elevated from baseline at 1 week postiron infusion with a mean increase of 455% (range 74%‐1189%), and elevated levels persisted until week 5 postinfusion. It is possible that FGF‐23 levels were even higher prior to the 1‐week measurement, as these levels were the peak measurements recorded. Similar to the results of Shimizu et al,^6^ 1,25(OH)_2_D levels postiron infusion were significantly lower compared with baseline.

FGF‐23 is produced primarily by osteocytes and is important for the regulation of 1,25(OH)_2_D and phosphate metabolism.^9^, ^10^ A lack of FGF‐23 results in hypophosphatemia and elevated 1,25(OH)_2_D levels, which has been demonstrated in FGF‐23 knockout mice.^10^ Conversely excessive FGF‐23, as occurs in autosomal dominant hypophosphatemia rickets, results in low 1,25(OH)_2_D and hypophosphatemia.^9^ It is not clear how FGF‐23 levels become elevated in response to intravenous iron treatment, but upregulated synthesis or secretion, reduced clearance, reduced proteolytic cleavage, or post‐translational modification of the cleavage site may be involved.^8^ Elevated C‐terminal FGF‐23, without elevation of intact FGF‐23, has been observed in patients with low serum ferritin, supporting the role of iron as a regulator of enzymatic cleavage of FGF‐23 in osteocytes.^11^, ^12^ Furthermore, hypophosphatemia has observed after administration of iron carboxymaltose in an small open‐label prospective RCT comparing iron carboxymaltose and iron dextran in 39 women with iron deficiency secondary to menorrhagia, while it was not observed in any of the patients receiving iron dextran, suggesting that the carbohydrate moiety and not just the iron element, may have an effect on FGF‐23 regulation.^12^ Independent of its effects on phosphate regulation and 1,25(OH)_2_D levels, FGF‐23 may also have direct detrimental effects on bone,^13^, ^14^ thus although phosphate and 1,25(OH)_2_D supplementation may be useful to normalize serum levels in the short term, ongoing use of intravenous iron should be avoided in patients with osteomalacia.^8^

Despite profound hypophosphatemia, urinary excretion of phosphate has been demonstrated to be inappropriately high in these cases of hypophosphatemia following use of saccharated ferric oxide, with reduced percentage tubular reabsorption of phosphate.^5^, ^6^, ^7^, ^8^, ^15^, ^16^ As a result, it has been hypothesized that since the saccharated ferric oxide molecule is small and neutrally changed, it is able to be filtered by the glomerulus and is directly toxic to the proximal tubular cells, possibly by causing peroxidation‐mediated damage to phosphate transporters.^7^ In contrast, the chondroitin sulfate‐iron particle is twice the diameter and is negatively charged, limiting its filtration through the glomerular basement membrane,^7^ and no cases of chondroitin sulfate‐iron–induced hypophosphatemia or osteomalacia have been reported to date.

In patients with nondialysis dependent chronic kidney disease (CKD), hypophosphatemia following iron infusion has been demonstrated to be rare and not reach clinically significant levels.^17^ It is therefore likely that renal transplant recipients are at increased risk of severe hypophosphatemia following intravenous iron due to preexisting secondary or tertiary hyperparathyroidism and exposure to medication that promotes phosphaturia such as calcineurin inhibitors.^15^

Most cases of iron‐induced hypophosphatemia may not be recognized unless patients become symptomatic or have blood tests shortly after the iron infusion. Blazevic and colleagues demonstrated that severe and symptomatic hypophosphatemia may persist for up to 6‐8 weeks in patients with preexisting mild hyperparathyroidism and vitamin D deficiency, including a renal transplant recipient and in a patient postbariatric surgery (Roux‐en‐Y)^15^ and another study has demonstrated that the duration of hypophosphatemia following treatment with ferric carboxymaltose can be as long as 9 months.^18^

In summary, hypophosphatemia is a frequent, but under‐recognized side effect following administration of intravenous iron, which can persist for weeks or months. The likely mechanism by which hypophosphatemia is induced is a directly toxic effect to proximal tubular cells, as well as elevated FGF‐23 and suppressed 1,25(OH)_2_D levels. Patients with preexisting hyperparathyroidism and vitamin D deficiency may be particularly prone, for example, patients who are post–kidney transplant or who have gut malabsorption. Conversely, patients with CKD or end‐stage kidney disease may actually be at reduced risk, due to reduced tubular phosphate excretion and preexisting chronically upregulated FGF‐23.^15^ Repeated iron infusions and associated hypophosphatemia can result in osteomalacia, and phosphate should be supplemented if there is severe, prolonged or symptomatic hypophosphatemia. We suggest that patients who have additional risk factors for hypophosphatemia, such as vitamin D deficiency and malabsorption, should have prospective monitoring of serum phosphate in the week after an iron infusion so that patients with severe hypophosphatemia can receive appropriate monitoring and phosphate replacement until phosphate levels normalize.

## CONFLICT OF INTEREST

The authors have no conflicts of interest to declare.

## AUTHOR CONTRIBUTIONS

MN was responsible for performing a literature search, drafting and revising the manuscript. RF was responsible for gathering case details, compiling investigation results, reviewing and revising the manuscript.
